# Platelet Transfusion Thresholds Among Children Admitted to a Pediatric Intensive Care Unit

**DOI:** 10.7759/cureus.1797

**Published:** 2017-10-24

**Authors:** Batool Alsheikh, Madhuradhar Chegondi, Balagangadhar Totapally

**Affiliations:** 1 Critical Care Medicine, Rady Children’s Hospital, San Diego, Ca; 2 University of Central Florida College of Medicine, Pediatrics, Nemours Children's Hospital, Division of Critical Care Medicine; 3 Dept. of Pediatrics, Herbert Wertheim College of Medicine Florida International University, Miami, Fl 33199, 4division of Critical Care Medicine and Nicklaus Children’s Hospital, Miami, Fl 33155

**Keywords:** platelet, threshold, transfusion, children, picu

## Abstract

Objective

To evaluate the threshold for platelet transfusion in children admitted to a pediatric intensive care unit (PICU). This is a retrospective chart review study, conducted at our tertiary level PICU and is related to critically ill pediatric patients who required platelet transfusion.

Methods

We retrieved the target patient population data from our blood bank database. The patients were subdivided into four subgroups: hematologic (hematologic malignancies, bone marrow suppression, hemolytic anemia, and sickle cell disease), surgical (post-surgical, trauma and acute bleeding), the unstable fraction of inspired oxygen (FiO_2_ > 0.6 and/or inotropic support), and the stable patients. Critically ill children between one month and 21 years of age were enrolled. We excluded patients who underwent extracorporeal membrane oxygenation (ECMO).

Results

A total of 197 transfusion episodes in 64 patients were analyzed. The distribution of transfusions episodes included hematologic 82% (n=161), surgical 7% (n=13), unstable 8% (n=16), stable 3% (n=7). The mean standard deviation (SD) of pre-transfusion platelet count (x1000) in all the patients and children in hematologic, surgical, unstable and stable groups were 29 (22), 29 (19), 47 (46), 28 (19), and 24 (14), respectively. The platelet count threshold for transfusion among the surgical group was higher compared to hematologic and unstable groups (p <0.001; analysis of variance (ANOVA) with multiple comparison tests). The mean platelet count during all episodes increased from 29 (22) to 71 (57) (p <0.05; paired t-test). The post-transfusion increase in platelet count was significantly higher among surgical and unstable patients compared to hematologic patients (p <0.001; ANOVA with multiple comparison tests).

Conclusion

The most common indication for platelet transfusion in the pediatric intensive care unit (PICU) is the underlying hematologic condition. The platelet count threshold for transfusion varied with the clinical condition and is higher among the surgical patients. The rise of platelet count after transfusion was higher among the surgical and unstable patients.

## Introduction

Given our new understanding of the various risks of transfusion of blood products, the general aim of the clinicians has been to limit their use. There are several known risks of transfusion of blood products, including transmission of viral and bacterial infections, hemolytic reactions, alloimmunization, transfusion-related acute lung injury (TRALI), transfusion-related immunomodulation (TRIM), and the volume overload in cardiac decompensated patients. Specifically, the platelets have a greater chance of bacterial contamination with either Staphylococcus aureus or Staphylococcus epidermidis when stored at room temperature. Despite routine testing for bacterial contaminants, platelet transfusions still have a contamination rate of 1:2000-3000 [1]. Compared to adults, children have an overall 2.6 higher rate of transfusion reaction (11.3/1000 vs. 5.29/1000 p-value <0.0001) [2]. According to Anglin, et al., the patients with traumatic brain injury with moderate coagulopathies who have received fresh frozen plasma (FFP) alone and in conjunction with packed red blood cells (PRBC) fared worse on cognitive and physical evaluations performed six months after transfusions [3]. Several studies have analyzed the effect of PRBC transfusions on multi-organ dysfunction. Specifically, the Transfusion Strategies for Patients in Pediatric Intensive Care Units (TRIPICU) study concludes that a lower hemoglobin transfusion threshold could be maintained in stable patients from three to 14 years of age without a significant impact on multi-organ dysfunction [4]. There has not been a randomized control study that has evaluated the platelet transfusion thresholds in critically ill children. Several factors need to be considered when approaching such a study including existing coagulopathies and consumptive platelet disorders. Also, the presence of sepsis and the need for further invasive surgeries should be considered [5]. The patients with hematological and oncologic diagnoses differ from unstable patients or those undergoing active bleeding. A platelet threshold of 100,000/mm^3 is advocated in the patients with traumatic brain injury, or those require neurosurgical procedures [1]. Ideally, one would isolate these critically ill pediatric patients in their subgroups to analyze thresholds, from there, one could then create a randomized control trial similar to the TRIPICU study and evaluate the risk of hemorrhage. Given the relative scarcity of dedicated pediatric platelet transfusion studies; however, the clinicians at times appear to transfuse at a wide range of thresholds, with many citing adult studies. To be able to change current practices and create future pediatric randomized controlled trial, we sought to perform an evaluation of current platelet transfusion practices in critically ill children over a two-year period, at a single tertiary care pediatric intensive care unit.

## Materials and methods

This study is a retrospective observational analysis of platelet transfusion thresholds in a single tertiary care PICU. After obtaining the Institutional Review Board approval, we obtained a list of all children admitted to the PICU during a two-year period. From our blood bank database, the list of the patients who received platelet transfusions was retrieved. The neonates and the patients requiring extracorporeal membrane oxygenation (ECMO), cardiac surgery and exchange transfusions were excluded. The children who received platelet transfusions outside of the PICU were also excluded from the analysis. For analysis, the patients were divided into four groups: hematologic, surgical, unstable and stable. The hematologic group included the patients with bone marrow suppression, hematologic malignancies, and hematologic diseases. The surgical group included patients who were post-surgical, trauma, and children with acute onset of bleeding without a known underlying hematologic condition. The unstable group included the patients receiving inotropic support and/or requiring the fraction of inspired oxygen (FiO2) greater than 0.6. The stable group included the patients who did not meet the criteria mentioned earlier. In a previous study, hemoglobin threshold for blood transfusion requirements was studied using similar groups [6]. Each patient’s demographics variables, pre- and post-platelet transfusion clinical variables and mortality were recorded. Transfusion variables included pre- and post-transfusion platelet count. Each transfusion episode was counted as an encounter if platelet count was measured before that transfusion. Multiple transfusions with no pre-transfusion platelet count were considered as a single transfusion encounter.

Statistical analysis

The descriptive data are expressed as the mean ± standard deviation (SD) for parametric data and median ± interquartile range (IQR) for nonparametric continuous data. The ANOVA test with multiple comparison-test was utilized to compare the means of all variables in four groups. A paired t-test was used to compare pre- and post-transfusion platelet values. A p-value of less than 0.05 was considered significant.

## Results

A total of 197 transfusion episodes in 64 patients (44% female) were analyzed. The ages ranged from one month to 21 years of age. The median age of our patient population was eight years (IQR two-15.5years). The distribution of platelet transfusion episodes included hematologic 82% (n=161), surgical 7% (n=13), unstable 8% (n=16), and stable 3% (n=7). The median PICU length of stay of all clinical groups was 14 days (IQR seven-23 days).

The mean pre-transfusion platelet count for all patients was 29X10^3 ± (22 x10^3). The mean pre-transfusion platelet thresholds for the subgroups were as follows: hematologic 28x10^3 ± (18.5x10^3); surgical 47x10^3 ± (46 x10^3); unstable 28x10^3 ± (19.2x10^3) and stable 24x10^3 ± (14x10^3). The post-transfusion platelet counts were as follows: hematologic 61X10^3 ± (43X10^3); surgical 148x10^3 ± (99x10^3); unstable 125x10^3 ± (77x10^3) and stable 60X10^3 ± (24x10^3) . The platelet count threshold for transfusion among the surgical patients was higher when compared to the hematologic and unstable groups, (p <0.001; ANOVA with multiple comparison tests as shown in Table 1). Accounting for all subgroups, the mean platelet count rose from 29x10^3 ±(22x10^3) to 71x10^3 ± (57x10^3) (p-value <0.05; paired t-test). Amongst the groups, the rise in the mean platelet count after transfusion was significant among the surgical and unstable patients as compared to the hematologic patients (P-value <0.001; ANOVA with multiple comparison tests).

**Table 1:** Demographic, hematologic, and transfusion variables in all children and children in various groups. *statistically significant p <0.001 when compared to surgical mean **Rate of rise statistically significant p <0.001 when compared to rate of rise of hematologic group.

	All children	Hematologic	Surgical	Unstable	Stable
Number of children	64	52	4	5	3
Number of transfusions	197	161	13	16	7
Median age in years (range)	8 (2-15.5)	5 (4-14)	6 (3-10.5)	2.8 (1.5-14.25)	16 (16-16)
Median length of stay in days (range)	14 (7-23)	21 (13-33)	13 (4-26.5)	12 (7.75-27.5)	26 (20-26)
Mean pre-transfusion platelet count X10^3/mm^3	29 ± 22	28 ± 18.5*	47 ± 46	28 ± 19.2*	24 ± 14
Mean post-transfusion platelet count X10^3/mm^3	71 ± 57	61 ± 43	148 ± 99**	125 ± 77**	60 ± 24

## Discussion

The mean platelet count threshold for transfusion in this study was 29X10^3 /mm^3. As expected, the platelet transfusion thresholds varied among the patients depending on the clinical condition. The platelet count threshold for transfusion was higher in the surgical cases when compared to the unstable and hematologic cases. This finding can be explained by analyzing the types of surgeries these patients were undergoing: intracranial bleeds, brain tumor resection, orthopedic surgeries, and other solid tumor resections. Besides, most of these patients had acute blood loss from the surgical site. In general, a platelet threshold of 50x10^3/mm^3 was used for the major surgeries, which may also explain the higher platelet threshold in the surgical patients with acute bleeding [7]. With regard to the hematologic subgroup, our observed threshold was close to the recommended threshold of 20x10^3 /mm^3, as proposed by initial recommendations from The Consensus Conference on platelet transfusion in 1987 [8]. However, this trigger has been challenged by several randomized control trials in adults [9-10]. There is a paucity of data in regards to current platelet transfusion thresholds in the PICU; however, there have been multiple randomized controlled trials conducted in the adults with hematologic and oncologic conditions from which one can extrapolate data. Bercovitz, et al. proposed that a platelet trigger of 10x10^3/mm^3 in stable hematologic patients can be implemented, although additional pediatric randomized controlled trials (RCTs) are needed to confirm [11].

The standard prophylactic platelet threshold of 20 X10^3 was challenged by Gmur, et al. in their study of the adult patients with acute leukemia [12]. This study proposed that the prophylactic platelet threshold can be lowered to 5x10^3/mm^3 with strict observation of the clinical signs of bleeding, the presence of fever, etc. The Platelet Transfusion Trigger Trial by Rebulla, et al. found no significance in the rates of the major bleeding between the two platelet prophylactic thresholds of 10x10^3 vs. 20x10^3 /mm^3 (p-value 0.41), in adolescents and adults undergoing induction chemotherapy for acute lymphocytic leukemia (AML) [13]. Similar results were found in the patients undergoing hematopoietic stem cell transplant [14]. In this study, when the patients were subdivided into 10x10^3 vs 20x10^3/mm^3 platelet transfusion thresholds, there were no differences in the incidence of the minor or major bleeding [14]. In another RCT, both the adult and pediatric patients were randomly assigned to transfuse 1.1, 2.2, 4.4 X10^11 platelets per square meter body-surface area at a threshold of less than 10,000/mm^3 [15]. This study concluded that rates of bleeding based on World Health Organization (WHO) criteria did not significantly differ amongst these various doses of platelet transfusions. These results provide evidence that the threshold for prophylactic transfusions in the patients who are otherwise stable does not alter the incidence of clinically significant bleeding. A study performed in the United Kingdom and Australia throughout 2006-2011, further substantiated that a prophylactic transfusion threshold does not significantly alter grade of bleeding in the adults. In this randomized, open-label, non-inferiority trial, it was shown that the adults who received prophylactic platelet transfusions <10,000 had a 50% chance of having WHO grade two, three or four bleeding (p <.06), vs. 43% in the non-prophylactic group [16]. However, the bleeding began later and lasted fewer days for those who underwent prophylactic transfusions (p-value 0.02 and 0.004 respectively). An extensive Cochrane review of prophylactic platelet transfusions and the risk of bleeding concluded that a set prophylactic transfusion threshold does not hinder bleeding, and there is no evidence that it will affect the incidence of WHO grade four bleeding [17]. However, a sub-analysis of the pediatric population from the Prophylactic Platelet Dose Trial (PLADO) trial performed by Josephson, et al. suggested that the pediatric population had more days of bleeding than adults (three days vs one day in adults P <00.1) and that children had a higher risk of grade two bleeding across all age groups (86%, 88%, 77%, vs. 67% for 0-5, 6-12, 13-18 years of age vs. adults, respectively with p <0.001) [18]. The data in critically ill children is lacking and the mean threshold for critically ill children will be higher, even in the hematologic patients, due to high acuity of illness and higher potential for serious bleeding complications. Most of the studies, both in adults and in children, address the threshold for platelet transfusion among the oncologic patients, and these studies are not restricted to critically ill patients in an intensive care unit (ICU). Also, the threshold for platelet transfusion in other clinical conditions in acutely ill children are not considered. Our study describes a single center experience of platelet count threshold for transfusion in various clinical groups in acutely ill children admitted to an ICU.

The current platelet transfusion practices vary depending on location, underlying diagnoses, and severity of illness. According to a 10-year epidemiological cohort study in pediatric oncology patients, the patients with acute myeloid leukemia (AML) received the highest number of platelet and red blood cell transfusion with median platelet transfusion for all patients being 16X10^9/L (IQR, 10 X10^9- 23 X 10^9/L) [19]. A survey of the American Society of Pediatric Hematology/Oncology members showed significant variability in practice for specific clinical scenarios. For instance, 21% of the clinicians would transfuse the patient with a brain tumor with no bleeding with counts of 10 X10^9/L, 37% for a range of 15-20X10^9/l and 42% for the threshold of >25 X 10^9/L [20]. Findings in our study also confirm that the threshold for the platelet transfusion varies with the clinical condition of the patients.

Our study showed that the rate of rising of platelet transfusions was greatest amongst the surgical and unstable patients compared to hematologic and stable groups. The low platelet increment response in hematological patients may be related to the coexisting coagulopathy, lymphocytotoxic antibodies and other comorbidities, as shown in the Trial to Reduce Alloimmunization to Platelets (TRAP) study [21]. There were very few patient encounters in the stable group. The results from our study provide a snapshot of current practices for various clinical conditions in the PICU. This information can be ideally used for future randomized control trials in which different platelet transfusions are implemented and its effects on bleeding and stabilization.

Limitations

The study had several limiting factors. First, the overwhelming majority of the patients that received platelet transfusions were hematologic, so one could argue that the overall mean was not an accurate representation of the average but an average of hematologic cases.  Within the hematologic subgroup, there was no delineation between those undergoing hematopoietic stem cell transplant or febrile neutropenic patients, who require higher thresholds secondary to the threat of sepsis. Also, incomplete data retrieval secondary to missing laboratory values resulted in the unavoidable elimination of those data sets. Furthermore, because of the incomplete records, we could not assess the time gap for post-transfusion counts after platelet administration. This being a single-center study, one needs to be careful in generalizing the findings from this study.

## Conclusions

We analyzed the current platelet transfusion practices in critically ill children admitted to our PICU. The most common indication for platelet transfusion in our PICU is the underlying hematologic condition. The mean pre-transfusion platelet count for all the patients was 29X10^3 ± (22 x10^3). The threshold platelet count for platelet transfusion varied with the clinical condition and was greater in children with surgical bleeding. In comparison to the various adult studies, the platelet transfusion threshold is higher in children admitted to a PICU.
